# Multicentre phase II pharmacokinetic and pharmacodynamic study of OSI-7904L in previously untreated patients with advanced gastric or gastroesophageal junction adenocarcinoma

**DOI:** 10.1038/sj.bjc.6603267

**Published:** 2006-08-01

**Authors:** S Falk, A Anthoney, M Eatock, E Van Cutsem, J Chick, H Glen, J W Valle, D W Drolet, D Albert, D Ferry, J Ajani

**Affiliations:** 1 Bristol Haematology and Oncology Centre, Horfield Road, Bristol BS2 8ED, UK; 2 Cookridge Hospital, Hospital Lane, Cookridge, Leeds LS16 6BB, UK; 3 Belfast City Hospital, Lisburn Road, Belfast BT9 7AB, UK; 4 University Hospital Gasthuisberg, Herestraat 49, Leuven B-3000, Belgium; 5 OSI Pharmaceuticals, Watlington Road, Cowley, Oxford OX4 6LT, UK; 6 Beatson Oncology Centre, Dumbarton Road, Glasgow G11 6NT, UK; 7 Christie Hospital, Wilmslow Road, Withington Road, Manchester M20 4BX, UK; 8 OSI Pharmaceuticals, 2860 Wilderness Place, Boulder, CO 80301, USA; 9 New Cross Hospital, Wednesfield Road, Wolverhampton WV10 0QP, UK; 10 UT MD Anderson Cancer Center, 1515 Holcombe Boulevard, Houston, TX, USA

**Keywords:** gastric/GEJ cancer, OSI-7904L, phase II, pharmacokinetics, pharmacodynamics

## Abstract

A two-stage Simon design was used to evaluate the response rate of OSI-7904L, a liposome encapsulated thymidylate synthase inhibitor, in advanced gastric and/or gastroesophageal adenocarcinoma (A-G/GEJA), administered intravenously at 12 mg m^−2^ over 30 min every 21 days. Fifty patients were treated. Median age was 64 years (range 35–82), 62% were male and 89% had ECOG PS of 0/1. A total of 252 cycles were administered; median of 4 per patient (range 1–21). Twelve patients required dose reductions, mainly for skin toxicity. Investigator assessed response rate was 17.4% (95% CI 7.8–31.4) with one complete and seven partial responses in 46 evaluable patients. Twenty-one patients (42%) had stable disease. Median time to progression and survival were 12.4 and 36.9 weeks, respectively. NCI CTCAE Grade 3/4 neutropenia (14%) and thrombocytopenia (4%) were uncommon. The main G3/4 nonhaematological toxicities were skin-related 22%, stomatitis 14%, fatigue/lethargy 10%, and diarrhea 8%. Pharmacokinetic data showed high interpatient variability. Patients with higher AUC were more likely to experience G3/4 toxicity during cycle 1 while baseline homocysteine did not predict toxicity. Response did not correlate with AUC. Elevations in 2′-dU were observed indicating target inhibition. Analysis of TS genotype, TS protein and expression did not reveal any correlation with outcome. OSI-7904L has activity in A-G/GEJA similar to other active agents and an acceptable safety profile.

Gastric cancer is one of the leading causes of cancer death worldwide, with an estimated 934 000 new cases diagnosed annually (Parkin *et al*, 2005). An estimated 22 400 new cases are diagnosed each year in the USA, with approximately 12 100 deaths in the same period (Jemal *et al*, 2003). In 2000, the estimated incidence and mortality in Europe was 88 002 and 71 022, respectively (Ferlay *et al*, 2001). In advanced disease, chemotherapy has been shown to improve survival and quality of life in comparison with supportive care alone (Glimelius *et al*, 1997). Phase II studies have demonstrated single-agent response rates of approximately 20% for a number of chemotherapeutic agents. However, responses are mainly partial and of a short duration, resulting in a median survival for patients with metastatic disease of around 6–9 months (Schoffski, 2002; Ajani, 2005).

Combination chemotherapy regimens have been developed in an effort to improve the outcome in advanced gastric cancer. Currently, ECF (epirubicin/cisplatin/continuous 5-Fluorouracil (5-FU)) or DCF (docetaxel/cisplatin/5-FU) combinations are considered but both have significant toxicities, such that many patients with advanced disease will not tolerate such treatment (Webb *et al*, 1997; Vanhoefer *et al*, 2000; Ross *et al*, 2002; Schoffski, 2002; Ajani, 2005; Moiseyenko *et al*, 2005).

OSI-7904 is a potent noncompetitive inhibitor of TS with a *K*_i_ value of 90 pM that does not require polyglutamation for maximal enzyme inhibitory activity (Smith *et al*, 1999; Schwartz *et al* 2001). OSI-7904 has been encapsulated into a liposomal formulation as OSI-7904L. This formulation increases plasma residence, tissue distribution and offers superior preclinical antitumour activity compared to parent drug or 5-FU (Desjardins *et al*, 2004). A phase I study with OSI-7904L utilising a day 1 every 21 days schedule determined 12 mg m^−2^ as the recommended phase II dose (Beutel *et al*, 2005). The toxicity profile was manageable and characteristic of other TS inhibitors (TSIs), including skin-related, gastrointestinal, fatigue and myelosuppression. Eleven of 31 patients with a variety of solid tumours achieved disease stabilisation despite extensive prior therapy, including other TSIs. Pharmacokinetic data from this trial demonstrated that in comparison to the nonliposomal drug, the liposomal formulation OSI-7904L had altered disposition properties and exhibited a longer plasma circulation time. Plasma levels of 2′-deoxyuridine (2′-dU) were found to be elevated two- to four-fold for 4–7 days, indicating that the target enzyme was inhibited following administration of OSI-7904L (Beutel *et al*, 2005).

Several groups have identified potential biomarkers, which might help predict outcome to TSIs in gastrointestinal malignancies. Firstly, Park *et al* (2002) studied the relationship of the promoter polymorphism in the TS gene – a 28 base pair tandem repeat DNA present in two or three copies – with outcome in patients with colorectal cancer who received capecitabine therapy. While a small retrospective pilot study, they showed that patients with a 2/2 polymorphism may have superior outcome to those patients with 2/3 or 3/3 genotype. They also reported that a 2/2 polymorphism was more likely to predict increased toxicity. Kawakami and Watanabe reported that a single nucleotide polymorphism (SNP) in the triple repeat can affect expression so that patients can be classified as either low or high expression type (Kawakami and Watanabe, 2003). It has also been shown that TS mRNA level and TS protein and gene expression can predict outcome to chemotherapy with 5-FU/leucovorin based therapy (Johnston *et al*, 1995; Lenz *et al*, 1995). In contrast, other authors suggest TS protein and/or expression are not major predicting factors for outcome to TSI-based therapy (Miyamoto *et al*, 2000; Tsujitani *et al*, 2000).

This study was performed to evaluate the antitumour activity of OSI-7904L in patients with advanced gastric or and gastroesophageal junction adenocarcinoma (A-G/GEJA). It was designed as a window study in order to test OSI-7904L in first-line patients without exposure to prior fluoropyrimidine therapy, and in the absence of clinical benefit, more commonly used combination regimens could be introduced without negatively impacting patient care. Additional biomarkers such as TS genotype, TS protein and expression were included as optional investigations in an attempt to identify potential predictive factors of outcome.

## PATIENTS AND METHODS

### Study design

This was an open-label, nonrandomised study to evaluate the antitumour activity of OSI-7904L in previously untreated patients with A-G/GEJA. The study complied with Good Clinical Practice and the Declaration of Helsinki, and was approved by ethics committees in all institutions prior to initiation. All patients gave written informed consent for the clinical study and separately for the genotype/biomarker study before trial entry.

### Patient selection

Patients with A-G/GEJA were enrolled provided they met the following criteria: age ⩾18 years; ECOG performance status ⩽2; estimated life expectancy ⩾3 months; no prior chemotherapy for advanced disease; no radiation therapy within 28 days prior to study entry; no concurrent anticancer therapy; adequate hematopoietic (neutrophils⩾1.5 × 10^9^ l^−1^ and platelets⩾100 × 10^9^ l^−1^); hepatic (bilirubin⩽1.5 times upper limit of normal (ULN) and serum AST and ALT⩽2.5 times ULN) and renal (serum creatinine⩽1.5 times ULN) function. Patients with symptomatic or unstable brain metastases or a history of serious illness were not enrolled.

### Treatment and dose modification

OSI-7904L was supplied by OSI Pharmaceuticals Inc. Drug was diluted in 5% dextrose in water and administered at 12 mg m^−2^ as a 30 min i.v. infusion repeated every 21 days.

Doses could be reduced to 9.6 mg m^−2^ and if required to 6.4 mg m^−2^ in case of: neutrophils <0.5 (× 10^9^ l^−1^) for ⩾7 days; febrile neutropenia; CTCAE grade 3 infection with neutropenia; platelets <25 (× 10^9^ l^−1^) or thrombocytopenic bleeding and/or CTCAE grade 3 nonhaematological toxicity. Any patient who failed to tolerate a second dose reduction was to be withdrawn. No routine premedication was required but any incidence of rash of CTCAE grade 3 severity could be addressed with either a dose reduction or by introduction of a steroid premedication at the Investigator's discretion.

### Study investigations

Baseline assessment included complete medical history, physical examination, vital signs, ECOG performance status, electrocardiogram, differential blood count and serum biochemistry (creatinine, urea, total bilirubin, alkaline phosphatase, AST, ALT, total protein and albumin). Baseline tumour burden was assessed by the appropriate radiological investigation (e.g. spiral CT scans). During treatment, assessments were repeated every three weeks while a complete blood count was carried out weekly (twice weekly in cycle 1). Investigator assessment of response was performed every two cycles according to RECIST criteria (Therasse *et al*, 2000). Adverse events were evaluated according to the National Cancer Institute Common Terminology Criteria for Adverse Events (NCI CTCAE) Version 3.0. Patients with disease progression were taken off study.

### Sample collection and analysis

#### Plasma pharmacokinetics

A total of seven blood samples were collected during cycle 1 and optionally in cycle 2: predose, 0.5, 4, 24, 48 or 72 and 168 h, then 22 days after the start of the infusion. Blood samples were collected in EDTA tubes and then processed and analysed for OSI-7904 concentration as previously reported (Beutel *et al*, 2005). Plasma concentrations *vs* time data for OSI-7904 were analysed by a noncompartmental method using WinNonlin version 4.1.b (Pharsight Corporation, Mountain View, CA, USA). Model 202 (i.v. infusion) was applied with linear/log trapezoidal rule.

#### Plasma 2′-dU

Four plasma samples (4–5 ml) were collected in cycle 1: predose, 24, 48 or 72 and 168 h after the end of infusion. Samples were collected into tubes containing sodium fluoride ethylenediamine tetra-acetic acid (NaF EDTA) and processed and analysed for relative 2′-dU concentration as previously reported (Beutel *et al*, 2005).

#### Plasma homocysteine levels

A blood sample (∼5 ml) was drawn from all patients prior to the OSI-7904L infusion on day 1 of cycle 1. Each sample was collected into an EDTA tube, immediately cooled on ice, and centrifuged at 1500–2000 **g** for 10 min under refrigeration within 30 min of collection. Equal aliquots of separated plasma were transferred into two labelled cryovials and frozen at −70°C. Homocysteine concentrations in plasma were determined using a fully automated Abbott Imx fluorescence polarisation immunoassay (Fiskerstrand *et al*, 1993).

#### Biomarkers

(i) TS/MTHFR genotype: A blood sample of 7–10 ml was collected into an EDTA tube and inverted several times with anticoagulant immediately after collection before storage at 4°C. DNA was isolated using the Puregene kit from Gentra Systems and genotyping performed using polymerase chain reaction amplification, using primers flanking the repeat region at the Department of Human Genetics at the University of Chicago. Amplified products were then sized on an agarose gel (Horie *et al*, 1995). Samples were processed to assess TS genotype (i.e. promoter polymorphism) and the SNP G → C within the promoter (Kawakami and Watanabe, 2003; Mandola *et al*, 2003). Similar methods were used to assess the MTHFR 677 C → T polymorphism (Skibola *et al*, 1999).

(ii) Immunohistochemistry (IHC) studies: Formalin fixed, paraffin-embedded tumour samples were received as 4–5 *μ*m sections on plus slides or 4 *μ*m sections were cut from the paraffin block provided. Immunohistochemistry staining was performed using the DakoCytomation Autostainer. Briefly, deparaffinised sections were treated with heat-induced epitope retrieval (HIER). A pH 9 retrieval solution (code S2367) was used for 40 min, followed by a peroxidase blocking (code S2001) step for 5 min. The TS antibody (code M3614) was used at a 1 : 50 dilution for 30 min followed by the EnVision+ labelled polymer anti-mouse System (code K4000) for 30 min. DAB substrate (code K3466) was used for 2–3 min. Slides were then counter-stained, cleared and coverslipped.

(iii) Gene expression studies: Quantitative determination of TS gene expression was assessed by RT–PCR from tumour samples and analysed relative to *β*-actin at Response Genetics Inc., Los Angeles, CA, USA (Schneider *et al*, 2004).

### Statistics

The primary objective of this study was to determine the objective response rate (ORR) of OSI-7904L in patients with untreated A-G/GEJA. The secondary objectives were to: determine the time to progression (TTP) and overall survival; determine the safety of OSI-7904L; further evaluate the PK profile of OSI-7904L; preliminarily evaluate any PK/PD correlation for safety and/or efficacy and investigate any correlation of genotypes and/or expression related to a patient's outcome.

An optimal two-stage design was used where the estimated response rate was based upon a low response rate of interest of 10% and a high response rate of interest of 25% (Simon, 1989). The study was calculated to have an *α*-error of 5% and a *β*-error of 20%. Under these assumptions, 18 evaluable patients were to be treated in Stage 1. At least three responses were required to continue in to Stage 2 when an additional 25 evaluable patients would be treated (up to a total of 43). If a total of eight responses or more were observed, the drug would be declared active.

## RESULTS

A total of 53 patients were enrolled into the study between October 2003 and June 2004 at 13 institutions in Europe and USA. Three patients deteriorated rapidly so that 50 patients received OSI-7904L. All 50 treated patients were evaluable for safety while 46 patients were evaluable for evaluation of response. Reasons for nonevaluability were: no measurable disease, disease not reassessed per protocol (two patients) and no disease reassessment. Patient characteristics are listed in Table 1.

A total of 252 cycles of OSI-7904L were administered, with a median of four per patient (range 1–21). Seventy-four percent (187 out of 252) were delivered at the planned dose of 12 mg m^−2^ with 21% (52 out of 252) at 9.6, 4% (10 out of 252) at 9.0 and 1% (three out of 252) at 6.4 mg m^−2^. Twelve patients had a dose reduction, mainly for skin toxicity (9 patients). Other reasons for dose reduction were (number of patients): stomatitis (2), diarrhea (2), fatigue (2), nausea (1) and vomiting (1), with some patients reporting more than one reason. Sixteen patients had cycle delays of which four were due to OSI-7904L-related toxicity. Despite these modifications, the median dose intensity and median relative dose intensities were 3.92 mg m^−2^ week^−1^ and 98.0%, respectively.

### Response

**Figure 1:**
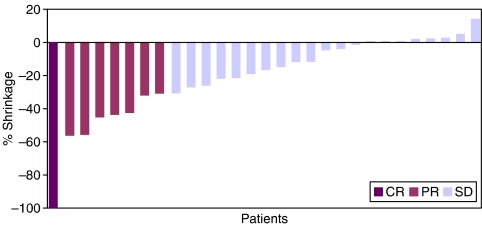
Illustration of percent tumour shrinkage in patients achieving response or stable disease.

Eight patients were classified by the investigators as obtaining a response according to RECIST (1 CR and 7 PRs). Thus, the investigator assessed response rate was 16% (95%CI: 7.2–29.1) in the 50 treated patients and 17.4% (95% CI: 7.8–31.4) in the 46 evaluable patients (Table 2). The median duration of response was 14.9 weeks (range 11.1–40.7). In addition, 21 patients (42%) achieved SD, including four patients with minor responses or unconfirmed partial response. The degree of tumour shrinkage in patients with CR, PR or SD is shown in Figure 1. In the evaluable population, the median TTP was 12.4 weeks (95% CI: 6.4–18.1). In the overall population, the median survival was 36.9 weeks (95% CI: 28.7–48.9) and 1-year survival was 31.8% (95% CI: 18.9–45.5). Twenty-five patients (50%) received second-line therapy (chemotherapy in 24 and radiotherapy in 1). The most common second-line regimen was ECF or some other platinum/TSI combination, with other patients receiving taxane- or irinotecan-based treatment.

All eight responses were among the 40 patients with gastric primary with 0/10 in those with GEJ. Similar comparisons for the same groups for TTP and overall survival were: TTP 17.6 weeks (95% CI: 11.4–22.1) *vs* 6.2 weeks (95% CI: 5.3–8.6) and 42 weeks (95% CI: 31.3–54.6) *vs* 18.65 weeks (95% CI: 13.10–41.7), respectively.

### Toxicity

The major haematological and nonhaematological toxicities reported in 50 patients receiving at least one dose of OSI-7904L are shown in Tables 3 and 4. The 30- and 60-day mortality was 2 and 12%, respectively. Cause of death was assigned to disease in all cases.

Severe haematological toxicity was uncommon with a single case of febrile neutropenia. The most common CTC grade 3/4 nonhaematological toxicities were skin-related with 22% of patients (11 out of 50) reporting symptoms, including rash, pruritis and erythema. Only one patient reported palmar-plantar erythrodysaesthesia. Dose reduction and/or introduction of some form of steroid premedication was found to ameliorate symptoms and permit continued treatment without recurrence of similar severity in the majority of patients. Other major CTC grade 3/4 toxicities included stomatitis/mucositis (14%), fatigue/lethargy (10%) and diarrhoea (8%).

### Pharmacokinetics and pharmacodynamics

Pharmacokinetic samples were collected from 48 patients in cycle 1 and five patients in cycle 2. Data confirmed a high level of interpatient variability with patients differing in the amount of drug cleared during the initial phase. Pharmacokinetic parameters (median: range) were determined by noncompartmental analysis: *C*_max_ 4.76 *μ*g ml^−1^ (2.35–20.5), terminal half-life 53.7 h (7.63–135) and AUC 123 h *μ*g ml^−1^ (9.65–383). Cycle 2 data demonstrated that there was low intrapatient variability with clearance consistent across repeat dosing (data not shown).

**Figure 2:**
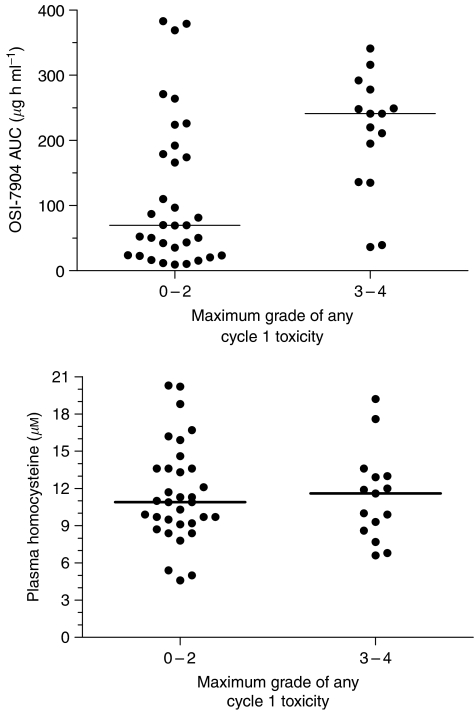
High plasma AUCs were associated with a greater risk of experiencing a grade 3 or 4 toxicity (upper panel) during dose cycle 1 (*P*=0.0049) while baseline plasma homocysteine concentration (lower panel) was not (*P*=0.8341). Bars represent median values.

Following the first OSI-7904L dose, plasma 2′-dU levels were elevated at least 1.5-fold in 24/27 (89%) patients with evaluable data indicating that the target enzyme was inhibited. Data indicated an increased risk of toxicity in patients with high AUC during cycle 1 (*P*=0.0049) (Figure 2). There was no relationship (*P*=0.8341) between baseline homocysteine and occurrence of severe toxicity during cycle 1 (Figure 2). Finally, no correlation was observed between AUC and investigator assessed response (*P*=0.6378, data not shown).

Plasma samples for TS genotype analyses were obtained from 37 (74%) patients. Seven (19%) were homozygous for the 2/2 repeat, 19 (51%) were 2/3 and 11 (30%) were 3/3. Analysis of the SNP in 34 patients further categorised these groups to high (16 (47%)) or low (18 (53%)) expression-type on the presence of the G → C transition. A total of 25 tumour samples (50%) were available for IHC analysis. These were stained with M3614 antibody and categorised based on intensity: 0 (3/25 (12%)), 1 (5/25 (20%)), 2 (10/25 (40%)) or 3+ (7/25 (28%)). Twenty (40%) samples were available for expression analysis and median level was 4.05 (range 1.02–11.10) relative to *β*-actin. There were no obvious correlations between the individual measures of TS. No significant increase in TS protein levels was detected by IHC in samples from two patients providing pre/post-treatment biopsies. There were no apparent correlations between TS genotype, SNP, IHC or expression in terms of TTP or grade of toxicity. Equally, there was no increase in TTP or toxicity for patients homozygous for the MTHFR 677 C → T polymorphism.

## DISCUSSION

This Phase II trial with OSI-7904L was designed as a window study in first-line A-G/GEJA to avoid prior exposure to 5-FU or other TSIs in the advanced setting. Patients could be switched to standard combination therapy in the absence of clinical benefit with single agent OSI-7904L. However, the median duration of treatment with OSI-7904L was four cycles and only one patient received alternative therapy prior to progression. Twenty-four patients (48%) crossed over to standard chemotherapy post progression despite often prolonged treatment with OSI-7904L demonstrating that OSI-7904L did not preclude second-line therapy in many patients. The ability to administer second-line therapy and overall survival similar to historical data suggest similar window designs are feasible to test new agents in gastric cancer (Webb *et al*, 1997; Ross *et al*, 2002). It was noted that the small subset of GEJA patients (*n*=10) appeared to have worse outcome than those with stomach tumours. This was an unexpected finding and contrasts with data from Ross *et al* (2002), who concluded that the different populations faired equally well with ECF treatment.

A second feature of this study was the collection of tissue and/or blood for assessment of potential biomarkers. Although shown to be feasible in this setting, the three different measures of TS (genotype, IHC and expression) all appeared to be of limited use in predicting outcome to OSI-7904L. The data generated showed no correlation between the different measures themselves and unlike other groups, failed to predict outcome based on any of the markers examined (Johnston *et al*, 1995; Lenz *et al*, 1995; Park *et al*, 2002; Kawakami and Watanabe, 2003). This may be attributable to the fact that the majority of tumour samples were retrieved from the original diagnostic block (Johnston *et al*, 2003). The lack of correlation between genotype and outcome in this study is similar to recent data from Sarbia *et al* (2006), who demonstrated that the MTHFR C677T and TS tandem repeat polymorphism had no predictive value in patients with oesophageal squamous cell carcinoma undergoing multimodality treatment.

The investigator assessed response rate of 17.4% is inferior to the 25% defined in the protocol and insufficient to reject the null hypothesis that the actual response rate could be less than 10%, defined as the cutoff for inactivity. However, the disease stabilisation rate demonstrates that many patients derived clinical benefit (Figure 1), receiving multiple cycles using a simple once every 21-day schedule with increased convenience. Other measures of efficacy, such as median TTP and survival of 12.4 weeks (95% CI: 6.4–18.1) and 36.9 weeks (95% CI: 28.7–48.9), respectively, also offer acceptable comparison to other specific and multitargeted TSIs, albeit in an uncontrolled manner (Schoffski, 2002; Ajani, 2005).

Nonhaematological toxicities were characteristic for this drug class: mainly skin, fatigue and gastrointestinal but there was a relatively low incidence of severe myelosuppression. A subset of patients experienced severe toxicity, and in addition to the 12 patients with reductions, five patients withdrawn during cycle 1 would have required a reduction had they continued on study, suggesting up to one-third of patients could not tolerate 12 mg m^−2^. However, dose reduction and/or dexamethasone prophylaxis appeared to ameliorate skin-related symptoms in most patients, facilitating repeat dosing. Unlike pemetrexed and plivatrexed, baseline homocysteine did not predict cycle 1 toxicity, suggesting that folate/vitamin B_12_ supplementation is not required or useful in preventing toxicity as it is with those agents (Niyikiza *et al*, 2002, Thomas *et al*, 2005).

The high level of interpatient variability in OSI-7904L clearance seen in the phase I trial of OSI-7904L was again evident (Beutel *et al*, 2005). Those patients with higher AUC were more likely to experience a CTC grade 3/4 toxicity (Figure 2). A similar observation was made between fluorouracil AUC and toxicity following administration of S-1 and cisplatin (Ajani *et al*, 2005). Ajani *et al* hypothesised that differential metabolism of the protective constituent (Oxo) or polymorphic differences in the *CYP2A6* gene as well as single dose PK may be important factors in attempting to assess the risk of toxicity with S-1 administration. In contrast, the utility of OSI-7904L may be hampered by the inability to predict the extent of exposure (due to the variability in initial clearance or alpha phase) and the fact that neither homocysteine nor genotype appeared to predict toxicity. The low intrapatient variability in PK suggests that monitoring blood levels following an initial lower dose of OSI-7904L might be a useful guide to longer term administration. There was no correlation between AUC and response, suggesting that OSI-7904L may not need to be administered at doses as high as 12 mg m^−2^. Beutel *et al* (2005) inferred that toxicity might not be the most appropriate predictor for dose assessment, suggesting some other pharmacodynamic marker may be considered such as 2′-dU. Unfortunately, while 2′-dU elevations were seen in this study, there did not appear to be any correlation with either toxicity or response.

In conclusion, the activity of OSI-7904L in A-G/GEJA appears borderline, though broadly similar to other agents, in terms of response rate, TTP and overall survival (Schoffski, 2002; Ajani, 2005). Overall the toxicity profile was manageable and administration with dexamethasone premedication is recommended to increase tolerability. The degree of disease stabilisation and duration of treatment are of note in this setting where disease palliation is a primary goal. The addition of a platinum agent might offer additional improvements in response or survival, similar to other TSI/platinum combinations (Schoffski, 2002; Ajani *et al*, 2005; Ajani, 2005). Preclinical data showing additive activity when OSI-7904L was combined with cisplatin or oxaliplatin have been reported and a phase I study combining OSI-7904L with cisplatin has been completed (Winski *et al*, 2004; Ricart *et al*, 2005). The dose of OSI-7904L was reduced to 7.5 mg m^−2^ in order to administer the drug safely in combination with 75 mg m^−2^ of cisplatin and may offer an improved therapeutic index compared to the single-agent dose of 12 mg m^−2^. Less skin toxicity was noted than that seen in these patients, probably as a consequence of the lower OSI-7904L dose as well as dexamethasone administered as antiemetic premedication for cisplatin. Despite the lower dose of OSI-7904L, responses were reported in three out of 27 patients, including a patient with gastric cancer (Ricart *et al*, 2005). Data from a study combining OSI-7904L and oxaliplatin are also awaited.

## Figures and Tables

**Table 1:** Patient characteristics

		**No. of patients (*n*=53^a^)**	**% of patients**
Male/female		33/20	62/38
Median age (range)	64 years (35–82)		
Gastric/GEJ		43/10	81/19
			
*ECOG PS*			
0		20	38
1		27	51
2		6	11
			
Median time from diagnosis (range)	58 days (13–1408)		
			
*Prior therapy*			
Surgery		37	70
Radiotherapy		5	9
Chemotherapy^b^		4	8
			
*Main sites of metastases*			
Lymph nodes		27	50
Liver		22	42
Lung		10	19

a Three patients were registered but deteriorated prior to dosing.

b Neo-adjuvant or adjuvant setting.

**Table 2:** Response rate

	**All treated patients (*n*=50)**			**Evaluable patients (*n*=46)**		
**Investigator best response**	***n***	**(%)**	**(95% CI)**	***n***	**(%)**	**(95% CI)**
Complete response	1	(2)	—	1	(2)	—
Partial response	7	(14)	—	7	(15)	—
Objective response (CR+PR)	8	(16)	(7.2–29.1)	8	(17.4)	(7.8–31.4)
Stable disease	21	(42)	—	21	(46)	—
Progressive disease	17	(34)	—	17	(37)	—
Not evaluable^a^	4	(8)	—	—	(0)	—

a Reasons for nonevaluable: no measurable disease, disease not reassessed per protocol (2 patients) and no disease reassessment.

**Table 3:** Incidence of haematological toxicity per patient by maximum NCI CTC grade

	**NCI CTCAE severity grade (total *n*=50)**					
	**Grade 1/2**		**Grade 3**		**Grade 4**	
**Haematological toxicity**	***n***	**(%)**	***n***	**(%)**	***n***	**(%)**
Total haemoglobin	40	(80)	6	(12)	1	(2)
Neutrophils	11	(22)	4	(8)	3	(6)
WBC	11	(22)	4	(8)	3	(6)
Platelet count	7	(14)	2	(4)	0	(0)

**Table 4:** Incidence of nonhaematological toxicity in ⩾10% of patients by preferred term and maximum NCI CTC grade

	**NCI CTCAE severity grade (Total *n*=50)**					
	**G1/2**		**G3**		**G4**	
**MedDRA system organ class total preferred term**	***n***	**(%)**	***n***	**(%)**	***n***	**(%)**
*Gastrointestinal disorders*	30	60	10	20	2	4
Nausea	29	58	2	4	0	0
Stomatitis	18	36	5	10	2	4
Diarrhoea	20	40	3	6	1	2
Vomiting	12	24	2	4	0	0
Constipation	5	10	0	0	0	0
						
*General disorders and administration site conditions*	34	68	6	12	1	2
Fatigue	19	38	2	4	0	0
Lethargy	10	20	2	4	1	2
Pyrexia	10	20	1	2	0	0
						
*Skin and subcutaneous tissue disorders*	28	56	10	20	1	2
Rash	20	40	8	16	0	0
Pruritus	19	38	1	2	0	0
Alopecia	11	22	—	—	—	—
						
*Metabolism and nutrition disorders*	20	40	3	6	0	0
Anorexia	18	36	2	4	0	0
						
*Infections and infestations*	10	20	1	2	1	2
Oral candidiasis	4	8	0	0	1	2
						
*Nervous system disorders*	10	20	0	0	0	0
Dysgeusia	6	12	0	0	0	0
